# Pleomorphic Hyalinizing Angiectatic Tumor (PHAT): Review of the Literature with Case Presentation

**DOI:** 10.3390/dermatopathology8020015

**Published:** 2021-04-04

**Authors:** Gerardo Cazzato, Anna Colagrande, Antonietta Cimmino, Teresa Lettini, Maria Teresa Savino, Carmen Martella, Giuseppe Ingravallo, Leonardo Resta

**Affiliations:** Department of Emergency and Organ Transplantation—Section of Pathology, University of Bari Aldo Moro, 70124 Bari, Italy; gerycazzato@hotmail.it (G.C.); anna.colagrande@gmail.com (A.C.); micasucci@inwind.it (A.C.); mariateresasa721@gmail.com (M.T.S.); carmela.martella@lifebrain.it (C.M.); giuseppe.ingravallo@uniba.it (G.I.); leonardo.resta@uniba.it (L.R.)

**Keywords:** soft tissue, PHAT, immunohistochemistry

## Abstract

Pleomorphic hyalinizing angiectatic tumor (PHAT) is a very rare entity of soft tissue considered a “neoplasm of uncertain behaviour of connective or other soft tissue” by the World Health Organization (2020). It develops in subcutaneous tissue of the lower extremities, more frequently in the region of the ankle and foot, and rarely as a deep-seated soft tissue mass in locations such as the perineum, buttock, arms, head and neck, and viscera. Although inconsistent cytogenetic data have been reported on PHAT so far, there are potential morphological and genetic overlaps with hemosiderotic fibrolipomatous tumor (HFLT) and myxoinflammatory fibroblastic sarcoma (MIFS). Here we report a case of PHAT at the level of the upper third of the right thigh in a 48-year-old patient and we also focus on the differential diagnoses of these entities and conduct a literature review of reported cases.

## 1. Introduction

Pleomorphic hyalinizing angiectatic tumor (PHAT) is a very rare entity of soft tissue, first described in 1996 by Smith et al. in a series of 14 cases as a “low-grade sarcoma of uncertain lineage” [1]. In “Soft Tissue and Bone Tumours” by the World Health Organization 2020, PHAT is considered a “neoplasm of uncertain behavior of connective or other soft tissue” [2] and is defined as “very rare” with, to our knowledge, about 100 cases reported in the international literature. PHAT affects patients from 10 to 79 years of age, with slight female predilection [3,4], and it develops in subcutaneous tissue of the lower extremities, more frequently in the region of the ankle and foot [4], and rarely as a deep-seated soft tissue mass in locations as perineum, buttock, arms, head and neck, and viscera [5]. Although inconsistent cytogenetic data have been reported [2] in PHAT so far, there are potential morphological and genetic overlaps with hemosiderotic fibrolipomatous tumor (HFLT) and myxoinflammatory fibroblastic sarcoma (MIFS) [2,4]. However, the link between these cancers remains controversial and not fully understood [2,3].

Here we report a case of PHAT at the level of the root of the upper third of the right thigh in a 48-year-old patient. We also focus on the differential diagnoses of these entities and conduct a literature review of reported cases. The targets were original articles and case reports of patients with histological diagnosis of PHAT. A comprehensive literature search was conducted using the PubMed database searching the following terms: “Pleomorphic Hyalinizing Angiectatic Tumor” and “Pleomorphic Hyalinizing Angiectatic Tumor of Soft Tissue”.

## 2. Case Report

A 48-year-old woman was referred to the U.O.C. of Plastic Surgery for a volumetric increase in a mass at the level of the right thigh, present for about 15 years and which in recent months had begun to cause functional discomfort. An echography (US) had confirmed the presence of an intensely vascularized subcutaneous lesion and in agreement with the patient it was decided to opt for the surgical option. The patient underwent a wide surgical excision with histologically confirmed margins free from neoplasm.

**Figure 1:**
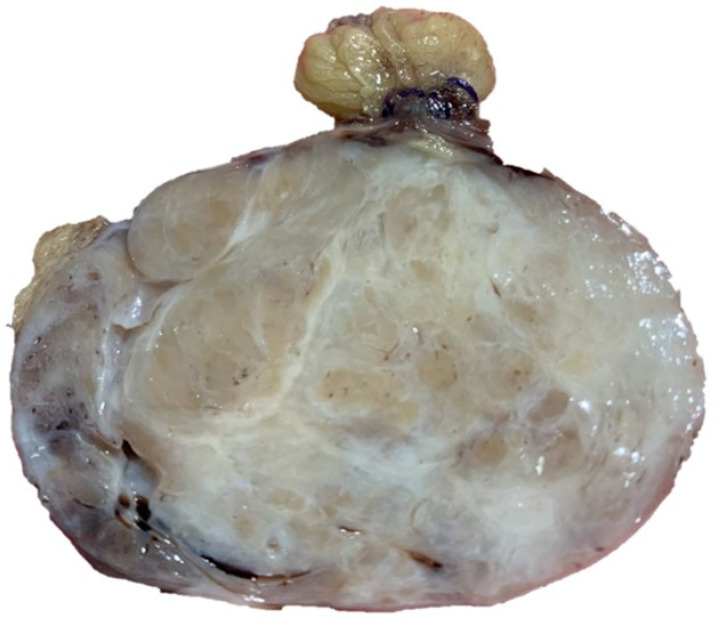
Collated appearance when cut and a greyish color.

The sample had therefore been sent to our laboratory and it appeared as a lesion of 6 × 5.5 × 5 cm, with a multiple chambered and collated appearance when cut and a greyish color (Figure 1).

After sampling, processing, inclusion in paraffin and microtome cutting, 5-micron thick sections were prepared and stained with routine staining (Hematoxylin-Eosin) and other sections were prepared by immunostaining with anti-CD34, Desmin, Vimentin, Actin Smooth muscle, Ki67 (MIB1), and S-100 protein. 

**Figure 2:**
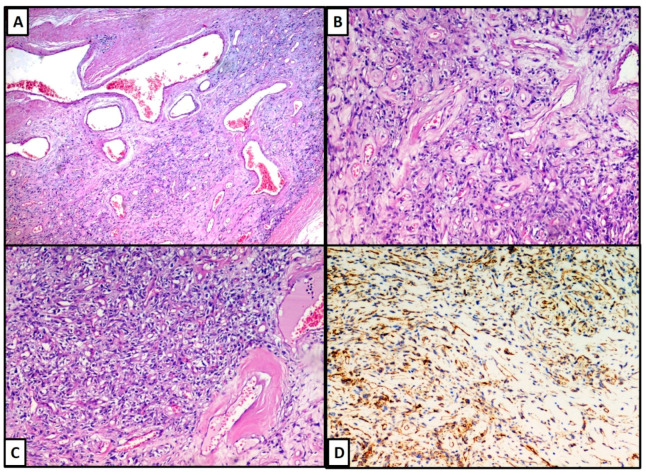
(**A**–**C**): The neoplasia was composted of clusters of variably sized, thin walled, ectatic blood vessels scattered and surrounded by a thick rim of amorphous eosinophilic material, with fibrosis phenomena. The lesion was positive for CD34 (**D**).

Microscopically, the lesion was composted of clusters of variably sized, thin walled, ectatic blood vessels scattered and surrounded by a thick rim of amorphous eosinophilic material, with fibrosis (Figure 2A,B). There were also organizing thrombi within blood vessels and the tumour cells were arranged in fascicles or, less frequently, sheets with spindle morphology (Figure 2C), sometimes hemosiderin pigment, and nuclear pseudo inclusions. At the periphery, the lesion showed a pseudo infiltrative pattern of growth. There were not cytological atypia and mitotic figures. From the immunohistochemical point of view, we found that the lesion was positive for CD34 (Figure 2D) and Vimentin, while it was negative for Desmin, S-100 protein and smooth muscle actin. The fraction of neoplastic proliferation (valued by KI67) was <5%. 

After a second opinion consultation with a soft tissue expert pathologist, PHAT diagnosis was placed. At the follow-ups of 6 months and 12 months, the patient showed no local recurrence.

## 3. Discussion

Pleomorphic hyalinizing angiectatic tumors was firstly described in 1996 by Smith et al. [1] and, to date, about 100 cases have been reported in literature [6]. Table 1 details authors, year of publication of the case series/case report, gender, age, and location of the PHAT. 

The mean age at diagnosis was 54.5 ± 17.1 (range 10–89). Of the patients, 61% (60/102) were female. The most common location was the lower extremity in 77.3% (78/102) of the cases, but other localizations were possible such as perineum, buttock, arms, head and neck, and viscera [5]. The average dimensions were around 5.3 cm, with an exceptional case of 26.3 cm in male breast reported by Lee et al. in 2005 [16]. Interestingly, Wei et al. [26] and Chalmeti et al. [35] reported two cases of PHAT respectively at the level of the renal hilum and within the kidney itself, emphasizing the potential problem of confusing this entity with other neoplasms of kidney. Our data completely agrees with Rush et al. [41], who in a recent article in 2018 conducted a detailed review of the literature more focused on the surgical-oncological implications related to PHAT. The best therapeutic strategist is represented by large surgery with free margins [26,41]. The recurrence rate is around 30–40% of cases [2,26,41] when it is not possible to be surgically radical, and local relapses have been described [41], although distant metastases have never been reported [2,41]. Local radiotherapy has also been shown to reduce the rate of local recurrence [26,28,41].

In most of cases it has a lobulated appearance, with a gray to light brown cut surface. It is never encapsulated, and many of these lesions have diffusely infiltrative edges, although well-defined margins [7,8,9,10,11,12,13,14,15,16,17,18,19,20,21,22,23,24,25,26,27,28,29,30,31,32,33,34,35,36,37,38,39,40,41]. Sometimes, PHAT can have a prominent cystic component [16,17,18,19]. From a histological point of view, PHAT is composed of clusters of ectatic blood vessels, surrounded by a thick rim of amorphous eosinophilic material, often with associated fibrosis. Immunohistochemically, PHAT is strongly positive for CD34, sometimes CD99 and negative for S-100 protein, Actin, Desmin, Cytokeratin, CD31, Factor VIII and Epithelial membrane antigen (EMA). Groisman et al. [9] reported immunoreactivity for vascular endothelial growth factor (VEGF), but this marker was negative in our case.

The morphological features of PHAT raised a wide range of differential diagnosis such as neurofibroma, schwannoma with ancient aspects, and undifferentiated pleomorphic sarcoma [7,11]. Although neurofibroma may show CD34 positivity, this entity is positive for S-100 protein and EMA, and the absence of cluster ectatic hyalinized vessel allows for a correct differential diagnosis. Unlike the ancient schwannoma, PHAT is encapsulated and it lacks Antoni A and B zones, as well as being negative for S-100 protein [5,6,7]. Finally, low mitotic count, lack of CD34 expression, tumor necrosis and intranuclear cytoplasmic pseudoinclusion can immediately exclude undifferentiated pleomorphic sarcoma [6,11,12,13,14].

Wei et al. [26] described the presence of unbalanced translocations involving chromosomes 1 and 3 and chromosomes 1 and 10 mapped to TGFBR3 and/or OGA (MGEA5) and in other related neoplasms such as myxoinflammatory fibroblastic sarcoma (MIFS): this feature led to the hypothesis that the origin of these tumors was similar and that neoplasms such as MIFS had acquired greater aggressiveness than PHAT and HFLT [30,41]. In spite of the small number of cases, this finding should be considered when following these patients and most importantly if a recurrence occurs [2,35,36].

## 4. Conclusions

We, herein, described another case of PHAT occurring in a middle-aged woman at the root of the right thigh.We have conducted a careful and detailed review of the literature but, considering the rarity of the lesion, future studies with large case series are needed to confirm or possibly deny the hypotheses of histogenesis formulated to date, and to further clarify which common precursor underlies the development of these particular types of lesions.

## Figures and Tables

**Table 1:** Case of PHAT reported in International Literature.

Author	Year	Number of pts	Gender	Average Age	Localization
Silverman, J.S. [7]	1997	1	F	59	Right foot
Fukunaga, M. [6]	1997	1	M	58	Axils
Gallo, C. [8]	1997	1	F	80	Right popliteal fossa
Groisman, G.M. [9]	2000	2	2 F	43	Lower extremities
Matsumoto, K. [10]	2002	1	F	83	Left thigh
Iwamoto, C.A. [11]	2003	1	F	49	Axils
Folpe, A. [12]	2004	40	22 F; 18 M	53	ankle/foot (n 15), leg (n 10), thigh (n 6), other (n 9)
Fujiwara, M. [13]	2004	1	M	69	Back
Lee, J.C. [14]	2005	1	M	63	Right breast
Luzar, B. [15]	2006	1	F	47	Ankle region
Capovilla, M. [16]	2006	1	M	66	Right buttock
El-Tal, A.E. [17]	2006	1	F	60	Right foot
Kazakov, D.V. [18]	2007	1	F	76	Axilla
Ke, Q. [19]	2007	9	5 F; 4 M	46	Lower extremities (n 2), inguinal (n 2), waist (n 1), forearm (n 1), buttock (n 1), foot (n 1), chest wall (n 1)
Jaggon, J.R. [20]	2007	1	F	77	Left loin
Tallarigo, F. [21]	2009	1	M	75	Breast
Peng, H.C. [5]	2010	1	M	49	Right buttock
Cimino-Matthews, A. [22]	2010	1	M	46	Right calf
Parameshwarappa, S. [23]	2010	1	F	65	Upper limb
Illueca, C. [24]	2012	2	2 F	51	Back and eyelid
Choong, M.Y. [4]	2012	1	M	53	Inguinal area
Subhawong, T.K. [25]	2012	3	2 F; 1 M	51	Lower extremities (n 2), upper extremity (n 1)
Wei, S. [26]	2012	1	F	37	Back
Idrees, M.T. [27]	2012	1	F	72	Renal hilum
Rekhi, B. [28]	2013	1	F	63	Lower leg
Kuang, P. [29]	2013	1	F	35	Neck
Changchien, Y.C. [3]	2014	1	M	76	Upper arm
Felton, S.J. [30]	2015	1	M	61	Back
Morency, E. [31]	2015	1	F	55	Dorsum of foot
Brazio, P.S. [32]	2016	1	F	22	Forearm
Kane, P.M. [33]	2016	1	M	35	Hand
Chu, Z.G. [34]	2017	1	F	26	Retroperitoneal
Chalmeti, A. [35]	2017	1	M	50	Left calf region
Scalici Gesolfo, C. [36]	2017	1	F	61	Kidney
Jaramillo, C.J. [37]	2018	2	2 M	50, 72	Right buttock (n 2)
Szep, Z. [38]	2019	1	M	63	Left crura
Balasubiramaniyan, V. [39]	2019	1	F	30	Mesorectum
Kökoğlu, K. [40]	2020	1	F	33	Oral cavity
